# Association between Metabolic Syndrome and Musculoskeletal Status: A Cross-Sectional Study of NHANES

**DOI:** 10.1155/2024/7330133

**Published:** 2024-09-21

**Authors:** Yue Shi, Shuhan Li, Xiaolong Xie, Yue Feng

**Affiliations:** ^1^ Chengdu University of Traditional Chinese Medicine, Chengdu 610075, China; ^2^ Meishan Hospital of Traditional Chinese Medicine, Meishan 620000, China

## Abstract

**Objective:**

The metabolic effects of metabolic syndrome (MetS) on musculoskeletal metabolism are controversial. This study explored the effect of MetS on bone mineral density (BMD) and muscle quality index (MQI).

**Methods:**

Data from the NHANES database from 2011 to 2014 were extracted, and nonpregnant participants aged 45–59 years were included. The included data were first weighted by complex sampling, and then, the effect of MetS on BMD and MQI was analyzed using multifactorial linear regression. We then performed a stratified analysis by gender and BMI classification. Moreover, a mediation analysis of MetS on BMD was conducted, with MQI as a mediating variable. A propensity score matching analysis method with a complex sampling design was additionally performed to verify the stability of the results.

**Results:**

A total of 1943 participants were eventually included. After adjusting for covariates, the results of linear regression show that MetS is associated with elevated pelvic BMD (beta = 0.03; 95% CI = 0.01, 0.06; *P*=0.02) and reduced MQI, especially arm MQI (beta = −1.02; 95% CI = −1.27, −0.77; *P* < 0.0001). MetS is more associated with BMD in women, MQI in normal or heavyweight, and BMD in lightweight, according to stratified analysis. MQI explains the indirect effect of MetS on BMD (beta = 0.007; 95% CI = 0.003, 0.010).

**Conclusion:**

This study provides evidence that MetS elevates BMD and reduces MQI, and further, that there is a mediating effect of MQI on elevated BMD.

## 1. Introduction

Metabolic syndrome (MetS) refers to a cluster of conditions that include high blood pressure, elevated glucose levels, high triglycerides, and increased waist circumference. It is a growing public health problem, with an estimated one-third of the population in the United States having it [1]. MetS is not only a risk factor [2, 3] for developing heart disease, stroke, and type 2 diabetes, but it has also been related to an elevated risk of some forms of cancer [4, 5]. In addition to the physical effects, MetS may have psychological repercussions. Researchers have shown that those who have MetS are more prone to have mental health problems including depression [6, 7] and anxiety.

MetS is a complicated set of metabolic disease syndromes. The skeletal muscle system, which is likely the biggest endocrine organ in the body, also performs the potent role of secreting active chemicals [8, 9]. Therefore, MetS may be implicated in several musculoskeletal system illnesses. The skeletal muscular system is also the primary organ that maintains the body's fundamental structure and takes part in movement, both of which have a significant influence on one's quality of life. The occurrence of sarcopenia, osteoporosis, and fractures increases with age. The coexistence of sarcopenia and osteoporosis is known as dysmobility syndrome, and it renders the elderly vulnerable to falls and fractures, making it one of the top causes of disability and mortality in the senior population. It is unclear, nevertheless, whether MetS impacts bone and muscle.

There are a limited number of imaging studies investigating whether limb muscle quantity and quality change after the development of MetS [10, 11]. Obesity is connected with lower muscle mass because the extra weight strains the muscles and makes them less able to contract and relax. Insulin resistance may lead to muscle mass loss because the body is unable to correctly utilize glucose and hence cannot provide the energy required to maintain muscular tissue. Although certain mechanisms suggest that MetS is associated with lower muscle mass, the effect of MetS on muscle quality is unclear. The impact of MetS on bone mineral density (BMD) is hotly debated; some publications claim there is no effect, while other research studies have shown increases [12] or decreases [13] in BMD. There may also be variations in BMD across different bone sections [14]. MetS is connected with higher inflammation, which may lead to bone tissue destruction. Furthermore, metabolic syndrome is linked to an increased risk of developing osteoporosis because the body's capacity to absorb calcium and other minerals required for healthy bones is compromised.

This study explored the association between MetS and muscle and bone utilizing data from the NHANES database, through two indicators, muscle quality index (MQI) and BMD, being used to represent muscle and bone conditions, respectively.

## 2. Materials and Methods

### 2.1. Study Design and Participants

This research relied on data from the National Health and Nutrition Examination Survey (NHANES). NHANES is a study of various demographics and health concerns in the United States. We selected data from two year-cycles for analysis, including 2011-2012 and 2013-2014. All participants' data in these two year-cycles were extracted, retained for participants aged 45–59 years, and excluded for pregnant women. We obtained MetS, BMD, and MQI data for regression analysis as well as mediation analysis. The National Center for Health Statistics Ethics Review Board approved NHANES, and the most recent review was on August 24, 2022. All participants signed informed consent forms.

### 2.2. MetS Diagnosis

The new diagnostic criteria [15] identified by the International Diabetes Federation (IDF) and the American Heart Association/National Heart, Lung, and Blood Institute (AHA/NHLBI) consultation were used in this study. The diagnostic criteria include the following five items, any three of which are met to diagnose MetS: (i) a waist circumference of not less than 102 cm in men and 88 cm in women in the U.S. population; (ii) elevated triglycerides of not less than 150 mg/dL; (iii) reduced HDL-C of less than 40 mg/dL in men and 50 mg/dL in women; (iv) systolic blood pressure of not less than 130 mmHg and/or diastolic blood pressure of 85 mmHg; and (v) fasting blood glucose greater than or equal to 100 mg/dL. For the last four items, drug treatment is an alternate indicator.

### 2.3. BMD Measurement

The dual-energy X-ray absorptiometry (DXA) assessment on the NHANES offers nationally representative data on body composition. DXA scans offer bone and soft tissue measures for the whole body, including the arms and legs, trunk, and head. The pelvis, left and right ribs, thoracic spine, and lumbar spine were also measured in bone. DXA scans were given to survey participants aged 8 to 59 years. Females who were pregnant were not allowed to take the DXA test. We selected three representative bone sites for analysis: total BMD, lumbar spine BMD, and pelvic BMD.

### 2.4. MQI Calculation

MQI [16] is used to measure the quality of the muscles. It is defined as the ratio of muscle strength per unit of muscle mass. The arm or appendicular skeletal muscle mass was also assessed by DXA. Hand grip strength was evaluated using a Takei dynamometer. Participants stood with their arm down straight and their wrists in a neutral posture. They were instructed to squeeze the dynamometer as tightly as they could. With 60 seconds of rest in between each measurement, the test was performed three times for each hand (dominant and nondominant), with the highest value being utilized.

We focused on three aspects of muscle quality. Arm MQI is calculated using dominant hand grip strength and dominant arm skeletal muscle mass, appendicular MQI using dominant hand grip strength and appendicular skeletal muscle mass, and total MQI using the sum of dominant and nondominant hand grip strength and appendicular skeletal muscle mass.

### 2.5. Covariates

The covariates included three main areas of data, namely, demographic data (age, gender, ethnicity, education level, and family income), lifestyle factors (smoking, alcohol consumption, physical activity, healthy eating index (HEI), and dairy products intake), and body mass index (BMI).

Those who smoke fewer than 100 cigarettes in their lifetime are nonsmokers, those who smoke more than 100 cigarettes but are not current smokers are past smokers, and those who smoke sometimes or daily are current smokers.

The following classification is used for the degree of alcohol consumption: Heavy alcohol users are those who drink more than 4 drinks per day (more than 3 for women) or binge drink more than 5 days per month; moderate alcohol users are those who drink more than 3 drinks per day (more than 2 for women) or binge drink more than 2 days per month; and those who do not meet the above criteria are classified as nondrinkers.

Physical activity indicates the total physical activity hours. The types of physical activity include tasks around home or yard, muscle strengthening activities, and walking or bicycling.

Data for HEI and dairy products are calculated as the sum of two days of dietary data. HEI calculation refers to the 2015 version.

### 2.6. Statistical Analysis

Data with missing values for MetS, total MQI, and total BMD were eliminated immediately during the cleaning procedure. The random forest method was used to fill in the missing values for the remaining variables. Continuous data are characterized by mean and standard errors, whereas categorical data are expressed by frequency and percentage. Linear regression was used to analyze the relationships between MetS, MQI, and BMD, with results presented as *β* (beta) and 95% confidence intervals (CIs). After stratifying by gender or BMI classification, the regression analysis was repeated. We also conducted a mediation analysis of MetS on BMD, with MQI as a mediator. Prior to analysis, the data were weighted. Weights were produced for complicated sample designs using two-year sample MEC exam weights (WTMEC2YR) and the variables SDMVPSU and SDMVSTRA.

Furthermore, propensity score matching (PSM) analysis was performed to do sensitivity analysis and validate the findings' stability. All of the covariates mentioned above except BMI are matched variables. The balance statistics for the unmatched data were weighted by the sampling weights. The matched data balance statistics were weighted by the product of the sampling and matching weights. The statistical description and linear regression were repeated after reweighting the data using the new weights. The linear regression results after adjusting Model 2 and after PSM are compared to ensure that the findings are stable.

All results were deemed statistically significant at *P* value <0.05. Statistical analysis of all data was performed using R software (version 4.2.2) with the packages “nhanesR” (version 4.2.2), “MatchIt” (version 4.5.0), and “mediation” (version 4.5.0).

## 3. Results

### 3.1. Population Characteristics

**Figure 1:**
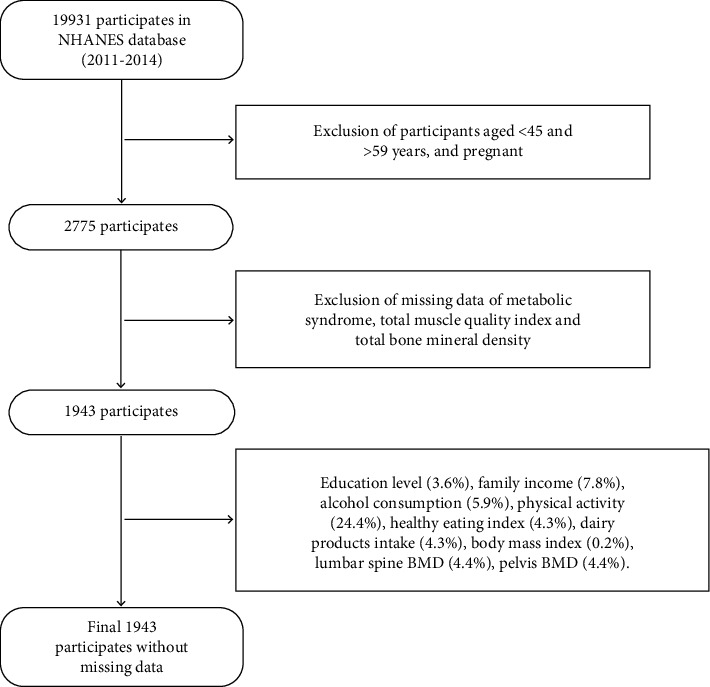
Flowchart for the participants' selection.

In the 2011-2012 and 2013-2014 year-cycles, there were 2775 participants aged 45–59 years (pregnant women were omitted). Missing data for MetS (0), total BMD (655), and total MQI (820) were subsequently deleted, and a total of 1943 individuals' data were included in this study, as shown in Figure 1.

As compared to individuals without MetS, those suffering from MetS have a somewhat lower household income, HEI index, and duration of physical activity. There are no variations in terms of age, gender, race, education level, or smoking. Baseline characteristics are detailed in Table 1.

### 3.2. Association of MetS with BMD and MQI

**Figure 2:**
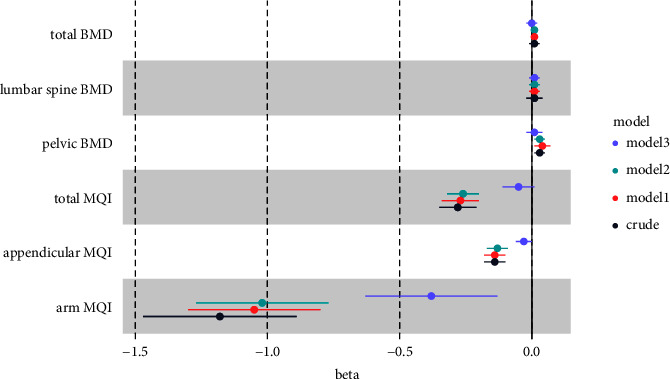
Associations of metabolic syndrome on bone mineral density and muscle quality index. Model 1: adjusted for age, gender, race, education level, and family income-to-poverty ratio. Model 2: further adjusted for smoking status, alcohol consumption, physical activity, healthy eating index, and dairy intake. Model 3: further adjusted for BMI.

Table 2 and Figure 2 illustrate the relationship of MetS with BMD and MQI. There is only a statistically significant increase in MetS and pelvic BMD, but no association with lumbar spine and total BMD. Independent of demographic and lifestyle characteristics, MetS is related to reduced muscle quality (Model 2). This link is decreased after additional adjustment of BMI, although MetS is still related to lower total and arm MQI. The effect sizes and 95% CIs of MetS for MQI and pelvic BMD are almost equal, whether adjusted for Model 1 or Model 2. MetS seems to have a somewhat steady impact on lowering MQI and boosting pelvic BMD. After further adjustment for BMI (Model 3), MetS was associated with lower arm MQI only, and the effect size was lower than that in Model 2.

### 3.3. Stratification Analysis

In the present study, stratified analysis was performed independently for BMI classification and gender, whereas Model 2 was adjusted. MetS is statistically significant for pelvic BMD in the BMI <25 stratum but not in the other BMI strata in the stratified analysis of BMI, as shown in Supplementary Table 1. Meanwhile, MetS has yet to discover an association between total and lumbar spine BMD. In the stratified analysis of gender, MetS is only found to be associated with pelvic BMD in women. MetS and MQI are found to be associated in both men and women; however, the effect is greater in women, as shown in Table 3. When female participants are stratified according to whether they were menopausal or not, higher BMD is detected exclusively in menopausal persons, and reduced MQI is reported in both menopausal and nonmenopausal women, while MQI falls more after menopause, which is shown in Supplementary Table 2.

### 3.4. Mediation Analysis

The results of logistic regression showed that arm MQI and pelvic BMD had the strongest effect in relation to MetS, so we selected arm MQI as a mediator of MetS on pelvic BMD for mediation analysis. The total effect of MetS on pelvic BMD is 0.03 (95% CI: 0.01–0.05), the indirect effect via arm MQI is 0.007 (95% CI: 0.003–0.010), and the percentage of mediating effect is 0.20 (95% CI: 0.08–0.56).

### 3.5. PSM Analysis

The distribution of the covariates is balanced between individuals with and without MetS for the reweighted data after PSM, and the results are shown in Supplementary Table 3. The linear regression results after adjusting Model 2 and after PSM are extremely comparable, as shown in Supplementary Table 4.

## 4. Discussion

This study shows that MetS is associated with increased BMD, particularly in the pelvis, in women; MetS is associated with decreased muscle quality in both men and women, but the magnitude of the effect is greater in women. The mediation analysis reveals that MQI explains 20% of the relationship between MetS and BMD.

The relationship between MetS and bone health reported in epidemiological studies is heterogeneous [17, 18]. The majority of studies found a correlation between MetS and decreased BMD [19–23], but some studies concluded that MetS increases BMD [24] or the two are not relevant. Results also vary widely by gender [25, 26] and by bone site [14]. For example, a meta-analysis [14] showed that MetS increased BMD of the spine, but not the femoral neck. The relationship between MetS and BMD was more evident in women, especially in postmenopausal women. In addition, the diagnostic criteria of MetS had an impact on the results [20]. Insulin resistance, adipose tissue, chronic inflammation, vitamin D insufficiency, oxidative stress, inflammatory cytokines, and mitochondrial dysfunction all play a role in the connection between MetS and sarcopenia [27]. MetS was associated with decreased muscle mass and quantity [28], and there was a linear dose-response relationship between decreased relative muscle strength and increased prevalence of MetS [29]. Lack of physical activity was a major risk factor for MetS and sarcopenia [30], but the association between MetS and MQI did not change markedly after adjusting for exercise variables. MetS may be associated with oligomuscular obesity, and the correlation between MetS and MQI in this study was almost reversed after adjusting for BMI, but we cannot guarantee the reliability of the results after adjusting for BMI due to the large difference in BMI distribution between MetS and non-MetS.

MetS disorders involving glucolipid metabolism in the body, such as insulin resistance, hyperglycemia, and hyperlipidemia [31, 32], may impact the activity and function of creatine kinase, as can inflammation caused by metabolic abnormalities. Creatine kinase is a family of enzymes that rapidly and reversibly transfer phosphate groups between ATP and creatine to produce creatine phosphate, a highly diffuse, high-energy phosphate molecule that plays an important role in muscle metabolism and has an irreplaceable and significant impact on muscle health and exercise capacity. It has been shown that mitochondrial creatine kinase governs mitochondrial phosphocreatine energy metabolism [33], which is required for osteoclast bone resorption, and that it is a key effector of osteoclast activation [34]. Osteoclasts are cells whose primary function is to break down aging bone tissue and promote bone remodeling, and if osteoclasts are inhibited, BMD may increase. It has been shown that low MQI shows a low CK response compared to high MQI [35], which decreases osteoclast activity and leads to increased bone mineral density. Due to the inability of old bone tissue to degrade and the lack of space for new bone tissue to develop, the microarchitecture of bone tissue is compromised, and despite an elevated BMD, bone health is compromised. Previous studies have found a positive correlation between lower BMD and sarcopenia, which may be due to confounding factors (e.g., dietary nutritional intake and physical activity). Exercise, for example, may improve bone and muscle mass. Exercise is a muscular loading stimulus that enhances creatine kinase synthesis and release during muscle metabolism. Meanwhile, exercise also regulates osteogenesis through various direct or indirect effects on bone cells.

The results also revealed that the impact of MetS on BMD and MQI was higher in women. According to one study, males exhibited greater levels of creatine kinase activity after exercise than women. This might be because males have greater muscular mass than women, while women have a higher body fat proportion. In addition, estrogen plays an important role in muscle growth and maintenance, maintaining muscle health by regulating protein synthesis, metabolism, and cell proliferation. Decreased estrogen levels may also lead to an increased risk of osteoporosis. MetS may indirectly affect muscle and bone health by affecting estrogen metabolism and synthesis. This may explain why the effects of MetS on muscle quality are more pronounced in menopausal women, which in turn is associated with bone health.

The findings emphasize the importance of incorporating BMD and MQI measurements into routine clinical evaluations of patients with MetS, which can serve as an early warning sign of potential skeletal and muscular complications, allowing healthcare providers to proactively identify and address problems. Second, clinicians can more customize treatment plans that include targeted interventions such as pharmacotherapy, dietary modifications, and exercise programs designed to improve bone density and muscle mass. The results of this study inform the development of public health policies aimed at prevention and early intervention. In addition, policymakers should consider incorporating these findings, which reflect the latest scientific evidence, into healthcare guidelines.

Future research should prioritize longitudinal studies to monitor the progression of MetS, BMD, and MQI, or Mendelian randomization studies to clarify causality. Second, it is recommended that studies be conducted in diverse populations, including individuals of different ages, genders, races, and lifestyles. In addition, multicenter and interdisciplinary collaborations are advocated to expand the scope and depth of research.

The current research offers certain benefits. First, this study is based on results from the NHANES database, which has a large study population and is weighted by a complicated sampling method to be representative of almost the whole U.S. population. Furthermore, this research establishes the impact of MetS on BMD and MQI, which was selected as a representative objective indicator to represent muscle and bone health. Of course, there are shortcomings with this study. First and foremost, since this is a cross-sectional study, the data cannot determine the causal relationship between the two, and more rigorous research is necessary to corroborate. Second, grip strength was measured in only two annual cycles, resulting in a limited amount of data. Third, this study was conducted on middle-aged Americans only, and the results cannot be directly applied to other races and other age populations.

## 5. Conclusions

This study shows that MetS is associated with increased BMD, especially in the pelvis, and decreased muscle quality; both of these effects were more pronounced in women. In addition, MQI explains 20% of the mediating effect in the relationship of MetS on BMD.

## Figures and Tables

**Table 1:** Baseline characteristics by metabolic syndrome.

	Total (*n* = 1943)	Non-MetS (*n* = 1258)	MetS (*n* = 685)	*P*
Age	51.88 (0.12)	51.48 (0.16)	52.64 (0.24)	<0.001
Gender				0.23
Female	979 (50.39)	636 (51.28)	343 (47.10)	
Male	964 (49.61)	622 (48.72)	342 (52.90)	
Family income	3.30 (0.10)	3.32 (0.11)	3.26 (0.10)	0.43
Race				0.18
Mexican American	213 (10.96)	132 (5.64)	81 (6.66)	
Non-Hispanic black	475 (24.45)	283 (10.18)	192 (12.77)	
Non-Hispanic white	777 (39.99)	513 (72.43)	264 (69.39)	
Others	478 (24.6)	330 (11.75)	148 (11.18)	
Education level				0.76
High school or equivalent	702 (36.13)	459 (32.42)	243 (33.12)	
College or above	1124 (57.85)	728 (63.70)	396 (62.16)	
Less than high school	117 (6.02)	71 (3.88)	46 (4.72)	
HEI^a^	52.08 (0.54)	52.84 (0.68)	50.65 (0.51)	0.002
Dairy intake	1.54 (0.04)	1.54 (0.05)	1.55 (0.07)	0.94
Smoke				0.03
Former	445 (22.9)	263 (23.04)	182 (31.13)	
Never	1041 (53.58)	697 (53.48)	344 (48.20)	
Now	457 (23.52)	298 (23.47)	159 (20.68)	
Alcohol consumption				0.15
Former	359 (18.48)	239 (16.28)	120 (15.77)	
Heavy	338 (17.4)	186 (17.00)	152 (21.74)	
Mild	641 (32.99)	436 (38.66)	205 (33.76)	
Moderate	322 (16.57)	211 (18.17)	111 (20.41)	
Never	283 (14.57)	186 (9.90)	97 (8.31)	
BMI	29.24 (0.27)	27.78 (0.29)	31.99 (0.42)	<0.0001
Physical activity	1074.71 (34.96)	1106.69 (45.16)	1014.08 (42.52)	0.11
Total MQI^b^	3.30 (0.03)	3.40 (0.03)	3.12 (0.04)	<0.0001
Arm MQI	12.34 (0.11)	12.75 (0.10)	11.57 (0.17)	<0.0001
Appendicular MQI	1.69 (0.01)	1.74 (0.01)	1.60 (0.02)	<0.0001
Total BMD^c^	1.10 (0.00)	1.10 (0.01)	1.11 (0.01)	0.21
Lumbar spine BMD	1.02 (0.00)	1.01 (0.01)	1.03 (0.01)	0.39
Pelvic BMD	1.22 (0.01)	1.21 (0.01)	1.25 (0.01)	0.02

^a^HEI: healthy eating index; ^b^MQI: muscle quality index; ^c^BMD: bone mineral density.

**Table 2:** Relationship of metabolic syndrome with bone mineral density and muscle quality index.

	Crude		Model 1^a^		Model 2^b^		Model 3^c^	
	*P*	*β* (95% CI)	*P*	*β* (95% CI)	*P*	*β* (95% CI)	*P*	*β* (95% CI)
BMD^d^								
Total	0.21	0.01 (−0.01, 0.03)	0.10	0.01 (0.00, 0.03)	0.11	0.01 (0.00, 0.03)	0.90	0 (−0.02, 0.02)
Lumbar spine	0.39	0.01 (−0.02, 0.04)	0.27	0.01 (−0.01, 0.04)	0.28	0.01 (−0.01, 0.04)	0.55	0.01 (−0.02, 0.04)
Pelvic	0.02	0.03 (0.01, 0.06)	0.01	0.04 (0.01, 0.06)	0.02	0.03 (0.01, 0.06)	0.40	0.01 (−0.02, 0.04)
MQI^e^								
Total	<0.0001	−0.28 (−0.35, −0.20)	<0.0001	−0.27 (−0.34, −0.20)	<0.0001	−0.26 (−0.32, −0.19)	0.09	−0.05 (−0.11, 0.01)
Appendicular	<0.0001	−0.14 (−0.18, −0.10)	<0.0001	−0.14 (−0.18, −0.10)	<0.0001	−0.13 (−0.17, −0.09)	0.10	−0.03 (−0.06, 0.01)
Arm	<0.0001	−1.18 (−1.47, −0.88)	<0.0001	−1.05 (−1.30, −0.80)	<0.0001	−1.02 (−1.27, −0.77)	0.01	−0.38 (−0.63, −0.13)

^a^Model 1: adjusted for age, gender, race, education level, and family income-to-poverty ratio. ^b^Model 2: further adjusted for smoking status, alcohol consumption, physical activity, healthy eating index, and dairy intake. ^c^Model 3: further adjusted for BMI. ^d^MQI: muscle quality index. ^e^BMD: bone mineral density.

**Table 3:** Relationship of metabolic syndrome with bone mineral density and muscle quality index, stratified by gender.

	Non-MetS	MetS	*P*
Total BMD^a^			
Female	Ref	0.016 (−0.001, 0.033)	0.063
Male	Ref	0.010 (−0.013, 0.032)	0.392
Lumbar spine BMD			
Female	Ref	0.028 (−0.002, 0.057)	0.065
Male	Ref	0.003 (−0.029, 0.034)	0.860
Pelvic BMD			
Female	Ref	0.037 (0.003, 0.071)	0.033
Male	Ref	0.030 (−0.004, 0.064)	0.079
Total MQI^b^			
Female	Ref	−0.303 (−0.384, −0.221)	<0.0001
Male	Ref	−0.211 (−0.321, −0.100)	0.001
Appendicular MQI			
Female	Ref	−0.154 (−0.200, −0.107)	<0.0001
Male	Ref	−0.111 (−0.174, −0.048)	0.002
Arm MQI			
Female	Ref	−1.25 (−1.628, −0.872)	<0.0001
Male	Ref	−0.818 (−1.210, −0.425)	<0.001

^a^BMD: bone mineral density; ^b^MQI: muscle quality index.

## Data Availability

The original data for this study are available from publicly available databases (https://wwwn.cdc.gov/nchs/nhanes/Default.aspx). Further inquiries can be directed to the corresponding author.

## Supplementary Materials

Supplementary Materials Supplementary Table 1: relationship of MetS with BMD and MQI, stratified by BMI categories. Supplementary Table 2: relationship of MetS with BMD and MQI, stratified by menopausal. Supplementary Table 3: baseline characteristics by MetS after PSM. Supplementary Table 4: comparison of linear regression results of the relationship of MetS with BMD and MQI before and after PSM.
