# Supplementary material for: Association between Metabolic Syndrome and Musculoskeletal Status: A Cross-Sectional Study of NHANES
Source: Int J Endocrinol. 2024 Sep 21;2024:7330133. doi: 10.1155/2024/7330133 (PMC11438510; doi:10.1155/2024/7330133)
Supplement: Supplementary Materials — Supplementary Table 1: relationship of MetS with BMD and MQI, stratified by BMI categories. Supplementary Table 2: relationship of MetS with BMD and MQI, stratified by menopausal. Supplementary Table 3: baseline characteristics by MetS after PSM. Supplementary Table 4: comparison of linear regression results of the relationship of MetS with BMD and MQI before and after PSM. [file 7330133.f1.docx]

**Supplementary materials**

Supplementary Table 1. Relationship of MetS to BMD and MQI, stratified by BMI categories.

|  | non-MetS | MetS | *P* |
| --- | --- | --- | --- |
| Total BMD^a^ |  |  |  |
| 25-30 | ref | -0.001(-0.031, 0.028) | 0.920 |
| <25 | ref | -0.001(-0.035, 0.034) | 0.971 |
| >=30 | ref | 0.002(-0.019,0.023) | 0.865 |
| Lumbar spine BMD |  |  |  |
| 25-30 | ref | 0.009(-0.033,0.052) | 0.648 |
| <25 | ref | 0.024(-0.036, 0.083) | 0.409 |
| >=30 | ref | 0.003(-0.032,0.038) | 0.849 |
| Pelvis BMD |  |  |  |
| 25-30 | ref | 0.016(-0.026,0.058) | 0.432 |
| <25 | ref | 0.057( 0.021, 0.093) | **0.004** |
| >=30 | ref | 0(-0.042, 0.041) | 0.981 |
| Total MQI^b^ |  |  |  |
| 25-30 | ref | -0.077(-0.141,-0.013) | **0.021** |
| <25 | ref | -0.012(-0.224, 0.201) | 0.907 |
| >=30 | ref | -0.040(-0.155, 0.075) | 0.470 |
| Appendicular MQI |  |  |  |
| 25-30 | ref | -0.040(-0.075,-0.005) | **0.029** |
| <25 | ref | -0.012(-0.140, 0.115) | 0.838 |
| >=30 | ref | -0.021(-0.080, 0.037) | 0.450 |
| Arm MQI |  |  |  |
| 25-30 | ref | -0.303(-0.614,0.008) | 0.055 |
| <25 | ref | -0.331(-1.406, 0.743) | 0.521 |
| >=30 | ref | -0.462(-0.870,-0.055) | **0.029** |

a BMD: bone mineral density; b MQI: muscle quality index.

Supplementary Table 2. Relationship of MetS to BMD and MQI, stratified by Menopausal.

|  | non-MetS | MetS | *P* |
| --- | --- | --- | --- |
| Total BMD^a^ |  |  |  |
| Menopausal | ref | 0.027( 0.004, 0.050) | 0.026 |
| Non-menopausal | ref | 0.005(-0.022,0.032) | 0.721 |
| Lumbar spine BMD |  |  |  |
| Menopausal | ref | 0.036(-0.003, 0.075) | 0.070 |
| Non-menopausal | ref | 0.037(-0.009, 0.082) | 0.107 |
| Pelvis BMD |  |  |  |
| Menopausal | ref | 0.046( 0.002, 0.091) | 0.042 |
| Non-menopausal | ref | 0.036(-0.008,0.080) | 0.102 |
| Total MQI^b^ |  |  |  |
| Menopausal | ref | -0.340(-0.451,-0.228) | <0.0001 |
| Non-menopausal | ref | -0.195(-0.381,-0.010) | 0.04 |
| Appendicular MQI |  |  |  |
| Menopausal | ref | -0.184(-0.248,-0.120) | <0.0001 |
| Non-menopausal | ref | -0.078(-0.172, 0.015) | 0.093 |
| Arm MQI |  |  |  |
| Menopausal | ref | -1.485(-1.987,-0.984) | <0.0001 |
| Non-menopausal | ref | -0.675(-1.293,-0.057) | 0.034 |

a BMD: bone mineral density; b MQI: muscle quality index.

Supplementary Table 3. Baseline characteristics by MetS after PSM.

|  | total | non-MetS | MetS | *P* |
| --- | --- | --- | --- | --- |
| Age | 51.89(0.13) | 51.78(0.18) | 52.08(0.28) | 0.42 |
| Gender |  |  |  | 0.74 |
| female | 979(50.39) | 636(50.25) | 343(51.77) |  |
| male | 964(49.61) | 622(49.75) | 342(48.23) |  |
| Family income | 3.29(0.10) | 3.30(0.11) | 3.27(0.11) | 0.72 |
| Race |  |  |  | 0.92 |
| Mexican American | 213(10.96) | 132(5.75) | 81(6.12) |  |
| non-Hispanic black | 475(24.45) | 283(11.35) | 192(11.40) |  |
| non-Hispanic white | 777(39.99) | 513(71.22) | 264(69.89) |  |
| others | 478(24.6) | 330(11.67) | 148(12.59) |  |
| Education level |  |  |  | 0.66 |
| High school or equivalent | 702(36.13) | 459(32.57) | 243(35.67) |  |
| College or above | 1124(57.85) | 728(63.01) | 396(60.02) |  |
| Less than high school | 117(6.02) | 71(4.42) | 46(4.31) |  |
| HEI | 52.23(0.58) | 52.39(0.70) | 51.93(0.71) | 0.58 |
| Dairy intake | 1.54(0.04) | 1.55(0.04) | 1.52(0.06) | 0.64 |
| Smoke |  |  |  | 0.48 |
| former | 445(22.9) | 263(24.59) | 182(27.74) |  |
| never | 1041(53.58) | 697(52.07) | 344(51.62) |  |
| now | 457(23.52) | 298(23.34) | 159(20.63) |  |
| Alcohol consumption |  |  |  | 0.82 |
| former | 359(18.48) | 239(15.93) | 120(17.48) |  |
| heavy | 338(17.4) | 186(17.94) | 152(19.06) |  |
| mild | 641(32.99) | 436(37.55) | 205(34.63) |  |
| moderate | 322(16.57) | 211(19.24) | 111(18.66) |  |
| never | 283(14.57) | 186( 9.35) | 97(10.18) |  |
| BMI | 29.21(0.33) | 27.78(0.30) | 31.81(0.54) | < 0.0001 |
| Physical activity | 1068.97(33.38) | 1078.96(38.54) | 1050.79(47.33) | 0.60 |
| Total MQI | 3.31(0.03) | 3.39(0.03) | 3.16(0.04) | < 0.0001 |
| Arm MQI | 12.35(0.12) | 12.69(0.09) | 11.74(0.21) | < 0.0001 |
| Appendicular MQI | 1.70(0.02) | 1.74(0.02) | 1.62(0.02) | < 0.0001 |
| Total BMD | 1.10(0.00) | 1.10(0.01) | 1.11(0.01) | 0.24 |
| Lumbar spine BMD | 1.02(0.01) | 1.01(0.01) | 1.03(0.01) | 0.24 |
| Pelvis BMD | 1.22(0.01) | 1.21(0.01) | 1.24(0.01) | 0.03 |

Supplementary Table 4. Comparison of linear regression results of the relationship of MetS with BMD and MQI before and after PSM.

|  | non-PSM (model 2) | |  | PSM (crude) | |
| --- | --- | --- | --- | --- | --- |
|  | *P* | β (95%CI) | *P* | | β (95%CI) |
| BMD |  |  |  | |  |
| Total | 0.11 | 0.01( 0.00,0.03) | 0.55 | | -0.01(-0.02,0.01) |
| Lumbar spine | 0.28 | 0.01(-0.01,0.04) | 0.39 | | -0.01(-0.04,0.01) |
| Pelvis | 0.02 | 0.03( 0.01,0.06) | 0.24 | | 0.01(-0.01,0.04) |
| MQI |  |  |  | |  |
| Total | <0.0001 | -0.26(-0.32,-0.19) | <0.0001 | | -0.31(-0.40,-0.22) |
| Appendicular | <0.0001 | -0.13(-0.17,-0.09) | <0.0001 | | -0.15(-0.20,-0.10) |
| Arm | <0.0001 | -1.02(-1.27,-0.77) | <0.0001 | | -1.09(-1.43,-0.76) |
